# Fireside Chats During COVID-19: Caregiver Community-Based Education

**DOI:** 10.1093/geroni/igaa057.3464

**Published:** 2020-12-16

**Authors:** Kara Dassel, Rand Rupper, Jorie Butler, Jacqueline Telonidis, Catherine Witt, Linda Edelman

**Affiliations:** University of Utah, Salt Lake City, Utah, United States

## Abstract

The Utah Geriatric Education Consortium (UGEC) provides education about Age-Friendly Health Care and Dementia-Friendly Communities to both informal and professional caregivers. As such, we have collaborated with our community partners to hold 12 “Fireside Chats” (2 in person, and 10 virtually due to COVID-19) between December 2019 and September 2020. Our expert speakers and panelists have given presentations on a variety of topics, specifically focused on coping with COVID-19, such as physical, music, and arts-based activities to do in the home, advance care planning, local services and supports, resiliency, and mindfulness. A total of 463 participants attended the Fireside Chats. A total of 169 attendees completed evaluations regarding the programs (a completion rate of 37%). Attendees were primarily White (86%), non-Hispanic (95%), well educated (86% had a college degree or higher), about half of the group were community caregivers (55%), while the other attendees were primarily from health professional backgrounds (e.g., nursing, social work, physical therapy). We obtained evaluation data in seven domains based on a 5-point Likert scale (1=strongly disagree to 5=strongly agree). The mean level of agreement in the seven following domains were: satisfaction (M=4.68/SD=.53), effectiveness (M=4.72/SD=.52), met stated goals (M=4.70/SD=.53), met educational needs (M=4.64/SD=.6), will improve the care they provide (M=4.57/SD=.70), included useful examples (M=4.59/SD=.66), and was better than similar trainings (M=4.23/SD=.86). These results along with steady attendance of our “Fireside Chats” demonstrate the need and satisfaction with our community-based education based on improving geriatric care practices within the community and in long-term services and support programs.

